# FALLS, FEAR OF FALLING, DAILY ACTIVITIES, AND QUALITY OF LIFE IN THAI COMMUNITY-DWELLING OLDER ADULTS

**DOI:** 10.1093/geroni/igad104.0784

**Published:** 2023-12-21

**Authors:** Jaroonrat Rodniam, Ladda Thiamwong

**Affiliations:** Panyapiwat Institute of Management, Sattahip, Chon Buri, Thailand; University of Central Florida, Orlando, Florida, United States

## Abstract

This study aimed to examine the prevalence of falls, fear of falling (FoF), activities of daily living (ADL) and quality of life (QoL) in community-dwelling older adults in Thailand. A cross-sectional survey of 433 community-dwelling older adults was conducted. We used a simple random sampling method to recruit urban and rural adults aged 60 years and older from 23 districts in Thailand. The measurements included demographics, fear of falling, history of falls, QoL, the modified Barthel ADL, chronic diseases, and medications. Data were analyzed by using both descriptive and inferential statistics. Among the participants, 45.3% reported having at least one fall in the past year, and 80% reported high level of fear of falling. Compared to non-fallers and persons without fear of falling, fallers and persons with fear of falling were more likely to have at least one chronic disease, hypertension and stroke. Fallers and persons with fear of falling were more likely to take more medications or anti-hypertensive drugs. Daily activities including eating, bathing, dressing, toileting, and walking differed significantly between non-fallers and fallers (p< 0.01 for all), and between persons with and without fear of falling (p< 0.01). Quality of life measures also differed significantly between fallers and non-fallers (p < 0.01). In conclusion, fallers and persons with fear of falling were more likely to have poorer health, more medications, lower quality of life, and different daily activity patterns. Future interventions should consider the differences between fallers and non-fallers and persons with and without fear of falling.

